# Transfer and Expression of Small Interfering RNAs in Mammalian Cells Using Lentiviral Vectors

**Published:** 2013

**Authors:** T. D. Lebedev, P. V. Spirin, V. S. Prassolov

**Affiliations:** Engelhardt Institute of Molecular Biology, Russian Academy of Sciences, Vavilova Str., 32, Moscow, Russia, 119991

**Keywords:** lentiviral vectors, shRNA, RNA interference

## Abstract

RNA interference is a convenient tool for modulating gene expression. The widespread application of RNA interference is made difficult because of the imperfections of the methods used for efficient target cell delivery of whatever genes are under study. One of the most convenient and efficient gene transfer and expression systems is based on the use of lentiviral vectors, which direct the synthesis of small hairpin RNAs (shRNAs), the precursors of siRNAs. The application of these systems enables one to achieve sustainable and long-term shRNA expression in cells. This review considers the adaptation of the processing of artificial shRNA to the mechanisms used by cellular microRNAs and simultaneous expression of several shRNAs as potential approaches for producing lentiviral vectors that direct shRNA synthesis. Approaches to using RNA interference for the treatment of cancer, as well as hereditary and viral diseases, are under active development today. The improvement made to the methods for constructing lentiviral vectors and the investigation into the mechanisms of processing of small interfering RNA allow one to now consider lentiviral vectors that direct shRNA synthesis as one of the most promising tools for delivering small interfering RNAs.

## INTRODUCTION

RNA interference is commonly used to inhibit gene expression. The advantages of this method include its simplicity, the possibility of quickly and significantly reducing the expression of any gene of interest, and the high specificity of the action. These properties render RNA interference a useful tool for investigating the role of specific genes in various cellular processes. For this purpose, entire libraries of siRNAs directed against a large number of genes have been created. Methods for applying RNA interference to the treatment of hereditary diseases, various neurodegenerative diseases, cancer, and as an antiviral therapy agent are currently under development. The search for new targets, the influence on which is efficient for treating a variety of diseases, is yet another application for RNA interference.

## RNA INTERFERANCE

**Fig. 1:**
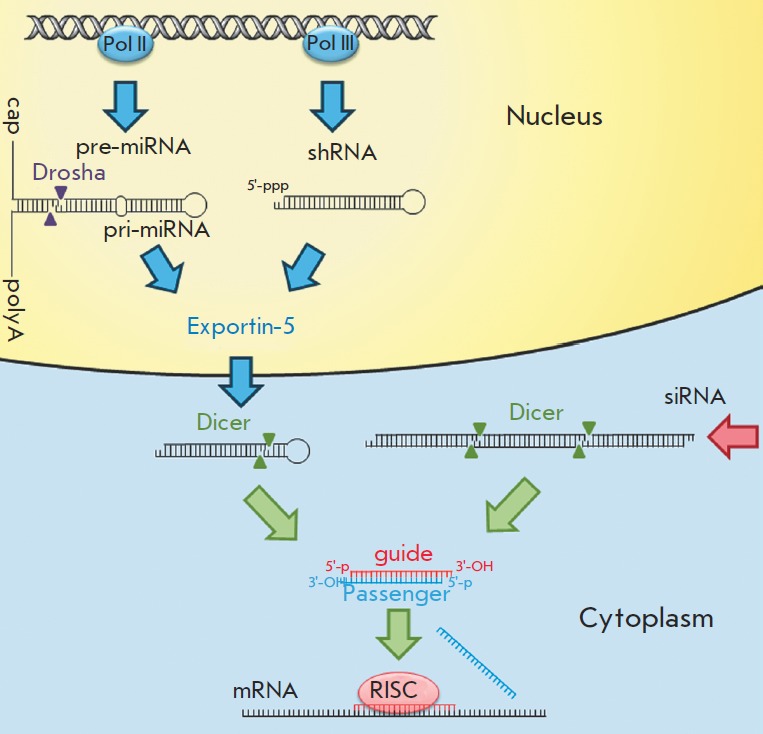
Cellular processing of small interfering RNAs. pri-miRNAs are transcribed by RNA polymerase II; the resulting transcripts undergo capping and polyadenylation. After these processes, pre-miRNAs are spliced out under the influence of Drosha (RNase type III). shRNA is transcribed by RNA polymerase III to form shRNA with a triphosphate at its 5’-end. Both hairpin structures (pre-miRNA and shRNA) are transported from the nucleus to the cytoplasm by the Exportin- 5 protein. In the cytoplasm, the Dicer protein splices out the sequences of future miRNAs and siRNAs from the hairpin structures and exogenous double-stranded RNAs. As a result of the processing by the Dicer protein, 21- to 25-bp-long RNA duplexes having a pair of unpaired nucleotides at their 3’-ends, OH-groups at their 3’-ends, and monophosphates at their 5’-ends are formed. The guide strand is loaded into the RISC protein complex, which binds to the mRNA that is complementary to the sequence of the guide strand. Meanwhile, the passenger strand is removed from the complex

RN A interference is a sequence-specific mechanism of suppressing gene expression, which is induced by the presence of exogenous or endogenous double-stranded RN A (dsRN A) in a cell [1]. This evolutionarily conserved mechanism functions in virtually all eukaryotic organisms. The sources of exogenous dsRN A include viruses or artificially introduced dsRN A. Endogenous dsRN A is formed as a result of the transcription of a cell’s own genes and often performs regulatory functions. The cleavage of long dsRN A by the Dicer protein, which belongs to the RN ase type III family (*Fig. 1*), resulting in the formation of small 21- to 25-nucleotide-long siRN A duplexes is the shared stage of all types of RN A interference. The duplex contains a pair of unpaired nucleotides and a pair of hydroxyl groups at the 3’-ends and monophosphates at the 5’-ends (*Fig. 1*). This structure of RN A duplexes enables their normal processing by a protein belonging to the Ago family, which plays a key role in the formation of the RISC complex (RN A-induced silencing complex) [2]. The RN A fragments formed as a result of Dicer-mediated cleavage of dsRN A are included in the structure of a RLC complex (RISC-loading complex) containing Dicer and TR BP proteins. During the next phase, the formation of a pre-RISC complex (complex preceding RISC) occurs. The structure of this complex includes the Ago-2 protein, which cleaves the RN A duplex, so that only the guide strand is retained in the complex [3]. This strand determines the specificity of expression suppression, while the other strand (known as the passenger strand) is removed from the complex [4]. The selection of the guide strand is independent of the prospective target; the strand whose 5’-end is characterized by a lower thermodynamic stability becomes the guide strand [5]. During the next phase, the guide strand forms a part of the RISC complex and binds to the site of the target mRN A according to the principle of complementarity (*Fig. 1*). The process of mRN A destruction involves two stages. First, a primary gap appears in the mRN A molecule, which is attributed to the endonuclease activity of the PIWI-domain of the Ago protein. This is followed by the destruction (degradation) of the target mRN A by cellular exonucleases [6]. If the complementarity of siRN A and mRN A is incomplete, the primary gap is not formed; hence, mRN A is not subjected to degradation. It is important to mention that even if the complementarity between the guide strand and mRN A is incomplete, suppression of gene expression can occur at the translation stage in a similar fashion to miRN A [7]. An alternative mechanism of siRN A action is associated with the formation of the RITS (RN A-induced transcriptional silencing) complex, which includes the Ago- 1 protein. The target mRN A is recognized by the RITS complex due to its interaction with RN A polymerase II during transcription [8]. During the next phase, the histone methyltransferases that methylate histones can become a part of the RITS complex, resulting in chromatin compaction and inhibition of the expression at the epigenetic level.

MicroRN As differ from siRN As by their mechanism of action and some features of their processing. Transcription of miRN As is carried out by RN A polymerase II. The resulting RN As undergo capping and polyadenylation [9]. Certain miRN As are encoded by individual genes, while others are encoded by entire gene clusters. miRN As can be transcribed together with mRN As; the sequence encoding miRN A is located in the intron of a protein-coding gene [7]. As a result of the transcription, pri-miRN A (miRN A precursor) is formed. Its structure includes a sequence of the future miRN A, the terminating loop, and flanking sequences [10]. Processing of pri-miRN As occurs in the nucleus with assistance from a complex consisting of two types of RN ase III, Drosha and DGCR 8 (in mammals). Approximately 65-bp-long hairpin-like miRN A precursors are formed as a result of the processing (*Fig. 1*) [11]. Transport of pre-miRN As to the cytoplasm is facilitated by the Exportin-5 protein (*Fig. 1*). In the cytoplasm, pre-miRN As are cleaved by the Dicer protein, resulting in the formation of an approximately 22-bp-long duplex [12]. Unlike the siRN A duplex, the miRN A duplex typically contains unpaired nucleotides in the middle. The miRN A is subsequently included in the RISC complex in a similar fashion to siRN A [13].

In contrast to siRN A, miRN A is usually fully complementary only to a small fragment of the mRN A (several nucleotides long). The miRN A fragment, which is completely complementary to mRN A, most frequently comprises the nucleotides 2–8 from its 5’-end and is known as a “seed region.” The “seed region” determines specific miRN A targets [14]. miRN A usually binds to the mRN A site, which is located in the 3’-untranslated region and is represented by multiple copies of the same mRN A. Since the length of the region that must be fully complementary is rather small, several different mRN As can act as targets for a single miRN A. It is presumed that full complementarity of miRN A and target mRN A can lead to mRN A degradation, while partial complementary binding of miRN A to mRN A can disrupt translation [7, 15].

**Fig. 2:**
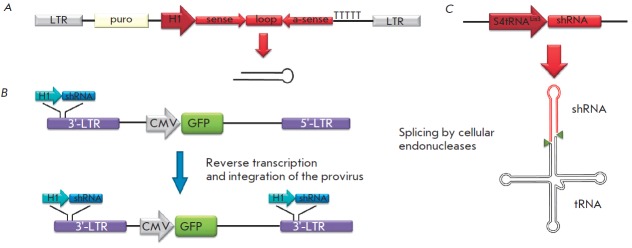
Schematic representations of the vectors that direct shRNA synthesis. A – The expression cassette is inserted between two LTR-sequences of the lentiviral vector. The expression of shRNA is directed by the H1 promoter. Transcription is initiated from the sequence of future siRNA (sense), followed by the “loops,” the inverted sequence, which is complementary to siRNA (α-sense), and termination sequence (thymines). The puromycin-resistance (puro) gene is also present in the vector; it enables the selection to be carried out [47]. B – The expression cassette is cloned into the 3'-LTR sequence. The cassette is doubled during reverse transcription; two copies of the expression cassette are formed following the integration of the provirus. A marker gene (e.g., green fluorescent protein (GFP) gene) under the control of the CMV promoter can also be cloned using this vector [45]. C – The expression cassette encoding shRNA fused with tRNA (S4tRNALys3-shRNA). After the transcription, the chimeric RNA undergoes processing in a similar fashion to normal tRNA, resulting in shRNA release [52]

The introduction of long dsRN As to mammalian cells induces interferon response; hence, short chemically synthesized siRN As are used. Their structure is similar to that of natural siRN As [16]. However, the effect of synthetic siRN As is short-term (only a few days), which is attributed to their degradation by cellular nucleases. Moreover, the concentration of these siRN As decreases during cell division. These drawbacks can be avoided if one uses vectors that direct the synthesis of siRN A precursors: small hairpin RN As (shRN Aa). shRN As contain the sequence of the siRN A guide strand (21–29 bp long), followed by a loop consisting of approximately 9 nucleotides, and a sequence that is complementary to the siRN A guide strand (*Fig. 2A*). The use of this structure enables to achieve long-term suppression of gene expression [17].

Lentiviral vectors are optimal tools for the delivery of shRN As into cells. An important feature of the life cycle of lentiviruses consists in their ability to integrate their genomes (in combination with proviral DNA) into cellular DNA. In addition, lentiviruses, as opposed to simple retroviruses, are capable of infecting nondividing cells. Despite the fact that such lentiviruses as the equine infectious anemia virus, feline immunodeficien- cy virus and the bovine immunodeficiency virus are used as templates for lentiviral vectors, the human immunodeficiency virus type 1 (HIV-1) remains the most commonly used virus for vector production. This is associated with the fact that the life cycle of this virus is better understood than those of the other viruses [18, 19].

## LENTIVIRAL VECTORS

Replication-incompetent systems based on lentiviruses are used for gene transfer and expression [20]. These systems enable the integration of the encoding target gene into the genome of the target cell’s DNA (transgene). A typical lentiviral vector contains *cis*-elements of the viral genome, which are required for the assembly and integration of the viral particle and the sequence encoding the target gene. All trans-elements of the viral genome are removed from the vector. Cotransfection of a vector and the plasmids encoding viral proteins is the main approach used for obtaining lentiviral vectors [19]. In order to reduce the risk of occurrence of replication-competent particles due to recombination, the components of the viral genome required for the assembly of lentiviral vectors are typically divided into three or four plasmids: one or two packaging plasmids, the vector plasmid, and the plasmid encoding the viral envelope protein. Constructs with all cis-elements (except for RRE and the splice donor site that is required for post-transcriptional processing of mRN A) removed are used today in third generation packaging systems. A heterologous promoter (usually CMV) and the polyadenylation signal of the SV40 virus are used instead of a long terminal repeat (LTR ). The* rev *and *gag/pol *genes are integrated into the cells using various expression cassettes. Humanization of the* gag/pol *genes is also employed, which enables their expression independently of *rev*. This also renders RRE removal from the packaging system possible [21]. It is important that such significant modifications of the packaging system do not affect the efficiency of the transduction by the lentiviral vector and significantly reduce the risk of occurrence of replication-competent particles due to homologous recombination. In order to reduce the risk of a nonhomologous recombination, a* trans*-lentiviral packaging system has been developed where the coding region of the *gag/pol *is divided into two parts and is incorporated into the structure of two different expression plasmids [22].

**Pseudotyping of lentiviral vectors**

In order to increase the tropism of lentiviral particles, the HIV-1 envelope protein is frequently replaced with the G protein of the vesicular stomatitis virus (VSV). These pseudotyped lentiviral particles enable the transduction of virtually all cell types. This modification not only expands the tropism of viral particles, but also increases their stability. Another important property of VSV-G is its ability to facilitate the penetration of the vector into the cell via endocytosis, thus reducing the need for auxiliary membrane proteins [23]. The main drawback of VSV-G pseudotyped lentiviral particles consists in their rapid elimination by the components of the immune system from the circulatory system [24].

One of the major problems encountered during the use of small interfering RN As is the insufficient specificity of their delivery into the target cells. In addition to VSV-G, heterologous glycoproteins of lyssaviruses, the lymphocytic choriomeningitis virus, alphavirus and baculoviruses can also be used to carry out pseudotyping [25]. The transduction efficiency of liver cells is increased with the use of the hepatitis C virus or baculovirus envelope proteins [26]. Pseudotyping of lentiviral particles by the envelope proteins of the Rabies virus enables the lentiviruses to infect the cells of the central nervous system *in vivo *[27]. The envelope proteins of other viruses are frequently used to ensure more efficient tissue-specific transduction.

Methods that enable the presentation of various cellular receptors and their corresponding antibodies on the surface of viral particles are becoming more common [28–30]. The general principle in this approach is to create a fusion protein which can be successfully integrated into the envelope of the vector particles to ensure a relative stability of these particles, on the one hand. On the other hand, this protein carries a fragment of the ligand required for binding to the receptor. Most frequently, this chimeric protein is based on a glycoprotein of the amphotropic murine leukemia virus (A-MLV) and the hemagglutinins of the influenza and measles viruses. These viral envelope proteins are modified in such a way that they can no longer recognize their natural receptors, thus avoiding nonspecific infection. Lentiviral vectors containing the epidermal growth factor (EGF) or an anti-CD20 single-chain variable antibody fragment (scFv) on their surface, which are fused with the hemagglutinin of the measles virus and intended for infecting B cells, have been produced based on this scheme [31]. Another approach consists in producing lentiviral particles containing the glycoprotein A-MLV fused with anti-CD3 scFv or with interleukin- 7 (IL-7), presented on their surface [32, 33]. This system enables the infection of T cells. Two ligands can be simultaneously used for pseudotyping: the stem cell factor (SCF) fused with the A-MLV glycoprotein and thrombopoietin (TPO) conjugated to the hemagglutinin of the influenza virus. Transduction of CD34+ cells with lentiviral particles carrying either thrombopoietin, or SCF, or both ligands on their surface, has proved significantly more efficient than the use of VSV-G as an envelope protein [34].

Utilization of viral surface envelope proteins is not the only way of presenting cell receptor ligands on the surface of viral particles. In this case, the utilized protein must contain a transmembrane domain; the surface of the viral vector must contain an envelope protein that can facilitate the fusion of the virus with the cell. Modified envelope proteins of the Sindbis virus or VSV-G, which have lost the ability to bind to their “native” receptor, are used for this purpose. The Sindbis virus has two surface envelope proteins, E1 and E2. The E1 protein is responsible for fusion with the cell, and E2 is responsible for binding to the receptor. The E1 protein functions independently of E2. A lentiviral vector containing the transmembrane form of SCF and a modified envelope protein of the Sindbis virus was produced according to this principle [35]. In the absence of the transmembrane domain, which is necessary for localization on the surface of lentiviral particles, the protein is attached to the transmembrane domain of VSV-G or human leukocyte antigen (HLA) [36]. For pseudotyping of lentiviral particles with antibodies, the packaging system must contain not only genes encoding light and heavy chains of antibodies, but also genes encoding Igα and Igβ proteins which are, required for antibody exposure on lentiviral particle surface.

This scheme was used to obtain lentiviral particles with surfaces containing anti-surface protein (CD20, DS-SIGN and CD3) antibodies [37–39].

Sindbis virus envelope proteins are also used for the pseudotyping of lentiviral particles by antibodies. For this purpose, the E2 protein is modified by incorporating the Fc-binding domain of protein A (ZZ-domain), which binds to immunoglobulin IgG, into its structure. Transduction using these lentiviral particles is only possible in the presence of monoclonal antibodies. The selection of antibodies determines the tropism of the lentivirus, enabling one to design viral particles that are specific with respect to cells of various origins without modifying the packaging system [40]. The disadvantages of Sindbis viral envelope proteins include the dependence of the protein E1 activity on pH (the pH value must lie within the range of 4.5–5.0). The reduced stability of these chimeric proteins during the pseudotyping of lentiviral particles can also be regarded as a drawback. The reduced efficiency of target cell infection using these lentiviral particles (which, however, can be compensated for by high specificity) should also be mentioned here.

Another approach that can provide specific infection of cells is the use of proteins as a component of the viral envelope, whose binding to a specific surface receptor results in a significant reduction in the efficiency of the transduction of those cells, the introduction of a transgene into which is undesirable [29]. This contamination-preventing protein can be bound to a viral glycoprotein using an amino acid sequence that is sensitive to certain proteases. The infection in this case involves two stages: first, the ligand on the viral surface binds to the cell receptor, and then cleavage of the peptide insertion occurs under the influence of certain proteases. After the insertion is cleaved, the glycoprotein can bind to its specific receptor on the cell surface. This approach enables to infect cells in the presence of specific proteases.

**The use of tissue-specific promoters**

Nonspecific cellular transduction, and therefore, transgene expression in these cells can cause a variety of adverse effects. In particular, transgene expression in antigen-presenting cells (APC) can result in the development of an immune response and T cell activation [41]. Tissue-specific promoters are used to reduce the effect of the nonspecific infection. Pseudotyping and the use of tissue-specific promoters enable to achieve transgene expression exclusively in the desired cells. However, tissue-specific promoters can be quite weak, and the level of expression of the target gene may be insufficient. The enhancers of stronger promoters can be used to strengthen these promoters. The enhancer of the CMV promoter used in combination with a variety of tissue-specific promoters provides a multifold increase in the expression of the target gene without decreasing the promoter specificity [42]. The site of transgene incorporation into the genome of the target cell is determined randomly; however, the incorporation takes place preferentially in the transcriptionally active regions. It is important to make an allowance for the fact that the incorporated transgene can accidentally come under the control of a strong promoter. In this case, its expression will be independent of the tissue specificity of the promoter. Insulators that block the effects of the neighboring enhancers are used to avoid this effect [28].

Transgene expression can be regulated at the posttranscriptional level. The mechanism underlying this regulation is based on RN A interference. Over 200 miRN As exhibiting tissue-specific expression have been identified thus far. It has been demonstrated that the introduction of four sites recognized by miR-142 miRN A, which is expressed mainly in hematopoietic cells, into the gene encoding the green fluorescent protein (GFP) reduces the level of fluorescence exclusively in these cells [28, 43]. Taking into account the fact that new miRN A expression patterns are continuously identified in various cells, it can be assumed that this method is of significant interest for precise control of the expression of the introduced genes.

**Small hairpin RNAs**

shRN As are siRN A precursors. They are typically expressed using U6 or H1 RN A polymerase III promoters (mouse or human) [43]. These promoters are small in size (about 400 bp long); transcription is initiated at the +1 position, and in the case of the U6 promoter it is desirable for the transcription to be initiated with guanine [44]. A sequence of 5–6 thymine residues acts as a transcription termination signal, resulting in the formation of double-stranded shRN A containing an unpaired 3’-end, which is essential for further processing by the Dicer protein. U6 and H1 promoters provide a stable and a relatively high level of shRN A expression in all cell types. shRN As obtained as a result of RN A polymerase III-mediated transcription have neither 5’- caps nor 3’-poly (A) sequences; they are not processed by the Drosha protein. Their transport to the cytoplasm is carried out by the Exportin-5 protein [12]. The use of the RN A polymerase III promoter during the production of lentiviral vectors that direct the synthesis of shRN A allows one to attain a high level of shRN A expression in virtually all cell types. There are approaches in which the cassettes expressing shRN A are cloned into the 3’-LTR region of a lentiviral vector [45]. During the synthesis of a provirus, 3’-LTR is used as a template for 5’-LTR . As a result, two copies of the expression cassette are incorporated into the proviral insertion (*Fig. 2B*).

The lentiviral vector frequently includes marker genes. Genes encoding fluorescent proteins or antibiotic resistance genes are typically employed. The presence of marker genes in a vector enables the selection of transduced cells and evaluation of the transduction efficiency. Lentiviral vectors that direct the synthesis of shRN A were used in the production of cell lines characterized by a stable suppression of the expression of the activated oncogenes detected in acute myeloid leukemias. The puromycin resistance gene was introduced into the vector as a marker gene (*Fig. 2A*) [46, 47].

When constructing the vectors that direct the shRNA synthesis, it is important to take into account the fact that the increased level of shRN A expression in cells may have adverse consequences. It was demonstrated that transduction of mouse hepatocytes using an adeno-associated virus-based vector, the shRN A transcription in which is controlled by the U6 promoter, results in liver lesions in 50% of cases [48]. A total of 49 different vectors, each encoding a unique shRN A, were used in the study [48]. The toxic effect of these vectors is associated with the competition between shRN As and cellular miRN As for interaction with the Dicer and Exportin-5 proteins involved in the processing of both types of small RN As. It is of significance that the resulting shRN A contains triphosphate at its 5’-end, which can cause an interferon response and stop the translation of cellular proteins. The presence of two unpaired nucleotides at the 3’-end of the shRN A stem is essential for efficient operation of the processing proteins (Exportin-5 and Dicer). An increase in the number of unpaired nucleotides significantly reduces the functional activity of these shRN As [49–51]. The formation of triphosphate at the 5’-end can be avoided using an approach characterized by simultaneous transcription of shRN A and tRN A [52]. The chimeric RN A processed by cellular endonucleases is synthesized, resulting in the formation of shRN A containing monophosphate at its 5’-end (*Fig. 2C*). The use of tRN A promoters allows one to prevent the emergence of nonspecific responses; the expression level of shRN As is considerably lower than when Polymerase III promoters are used.

If the first 2–8 nucleotides of the siRN A guide strand are complementary to the “seed region” of a particular miRN A molecule, then this siRN A molecule can function as a miRN A. This can trigger a nonspecific action from siRN A. The ability of siRN A to act as miRN A can be used to suppress the expression of certain genes (e.g., the CCR 5 gene) [53]. siRN A, which is specific with respect to the CCR 5 gene, is complementary to the “seed region” located in the 3’-UTR of mRN A. This siRN A caused the degradation of mRN A and resulted in disruption of translation in a fashion similar to the action of miRN A. When selecting shRN A sequences, one should bear in mind that the 5’-end of the guide strand of the duplex formed as a result of processing must be characterized by a lower thermodynamic stability. Inconsistency with these rules can result in the following: the passenger strand will become part of the RISC complex, instead of the guide strand, leading to a reduced specificity of the shRN A action. It is assumed that the H1 promoter is better suited for *in vivo *application as compared to the U6 promoter, since the H1 promoter is less toxic despite its lower efficiency [54]. Successful application of lentiviral vectors guiding the shRN A synthesis was demonstrated using animal models of various diseases [55–57]. In particular, the expression of shRN A persisted for 9 months following the injection of lentiviral particles, and suppression of the reporter gene expression in mouse brain cells was maintained [58].

**Fig. 3:**
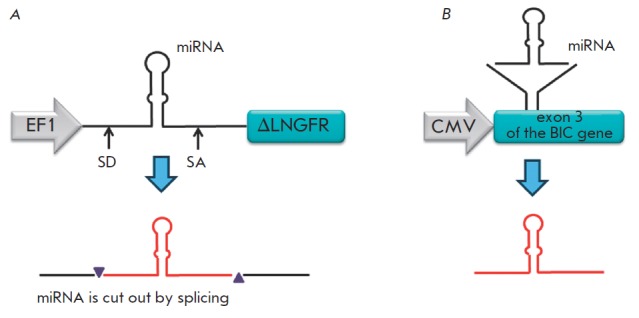
Schematic representations of the vectors that direct the synthesis of the modified miRNAs: A – miRNA is cloned under the action of the EF1’ promoter in such a manner that it is expressed along with a fragment of the NGFR(ΔLN GFR) gene in the first intron and is cut out by splicing. SD – splice donor site; SA – splice acceptor site [62]. B – miRNA is expressed as a component of exon 3 of the BIC gene, where it was originally present [63]

The adverse effects associated with the use of shRNAs (interferon response, competition with cellular miRN As, nonspecific action) can be avoided by employing various approaches. Several of them are based on the adaptation of the artificial shRN A processing to the mechanisms used by cellular miRN As [59]. To achieve this objective, the sequence of the guide strand of the future miRN A can be replaced with an artificial sequence, while conserving the structure of miRN A pre cursors. miRN As are transcribed by Polymerase II; thus, it is preferable to use the promoters of this enzyme when constructing the vector. It was demonstrated that expression of shRN A under the control of the U6 promoter in an adeno-associated virus-based vector is 10 times more efficient than the expression of miR-30 under the control of the same promoter. However, the suppression level of the reporter gene was approximately the same, while the toxic effect of the construct containing miR-30 was much lower [60, 61]. The fact that the Dicer protein can select both strands of the miRN A duplex is a drawback of miR-30-based systems. Cell transduction with lentiviral vectors carrying the gene for the nerve growth factor receptor (NGFR), whose first intron contains an integrated sequence encoding pri-miR-223 (200 bp) under the control of the integrated EF1α promoter, results in stable expression of the* NGFR *gene and miRN A (*Fig. 3A*). The sequence of the guide strand in the “stem” of miRN A can be replaced by other guide strand sequences from other miRN As or siRN As [62]. An approach enabling one to achieve stable expression of the mouse *BIC *gene and its miR-155 product characterized by an altered sequence of the guide strand has also been developed (*Fig. 3B*) [63]. The vector containing a fragment of the *BIC *gene (including the sequence of miR-155) directed the successful expression of both the source miRN A and miRN A with an altered sequence of the guide strand (*Fig. 3B*) [63]. General rules for constructing artificial miRN As have yet to be developed, which is primarily due to insufficient knowledge with regard to their processing.

**Simultaneous synthesis of several small interfering RNAs**

In some cases, the simultaneous expression of multiple siRN As is preferable (e.g., during antiviral therapy). This is attributed to the fact that some viruses mutate at a high rate, and the probability of developing resistance to specific siRN A among them is high. The use of multiplex constructs enabling the synthesis of several siRN As significantly reduces the probability of emergence of resistant forms of the virus.

**Fig. 4:**
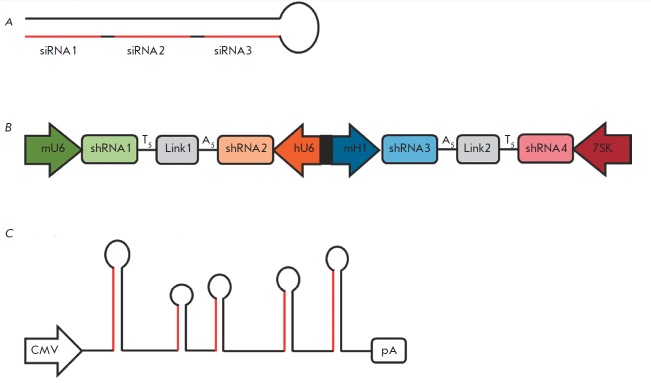
Various approaches to the multiplex expression of small interfering RNAs. A – long-hairpin RNAs (lhRNAs) contain sequences of several siRNAs (highlighted in red), which are subsequently spliced out by the Dicer protein [64]. B – Expression of four shRNAs from a single vector under the influence of various promoters of RNA polymerase III [68]. C – Expression of several miRNAs using mir-17-92 polycistron. miRNAs with altered sequences of the guide strands (highlighted in red) were integrated into the base of mir-17-92 polycistron [69]

Therefore, a lentiviral vector that directs the synthesis of long hairpin RN As (lhRN As) containing the “loopstem” structure was constructed. Processing of these lhRN As occurs with assistance from the Dicer protein; several siRN As are formed under the influence of the latter. The lhRN A (the precursor of siRN A) nucleotide sequence is selected according to the same principle as per shRN A selection process. Suppression of HIV-1 replication was achieved with assistance from the 50- to 80-bp-long lhRN A, which acts as a precursor for 2–3 siRN As against various parts of the general region of* tat/rev *genes, (*Fig. 4A*) [64]. A similar approach was used to suppress the replication of the hepatitis B and C viruses [65, 66]. The efficiency of lhRN A processing by the Dicer protein decreases as the siRN A sequence approaches the “loop,” resulting in the formation of various amounts of siRN A and a nonuniform suppression of target gene expression.

Since the promoters of RN A polymerase III are relatively small (200–400 bp), a single vector can incorporate several siRN A sequences, each controlled by its own promoter. Different RN A polymerase III promoters (U6, H1 and 7SK) are used in this case, since the utilization of identical promoters may induce recombination between their sequences and deletion of one or several expression cassettes in 80% of the cases [67]. A vector ensuring the synthesis of four shRN As under the control of the mouse U6, H1 and human U6, 7SK promoters has been constructed. In this case, the mouse H1 and human U6 promoters were fused into a single bidirectional promoter (*Fig. 4B*). Suppression of the expression of four different genes was achieved using this vector [68].

Clusters encoding polycistronic miRN As, which form several pre-miRN As, can be used for simultaneous expression of multiple siRN As. Transcription of the *miR- 17-92 *gene cluster gives rise to double-stranded primiRN As approximately 1 kbp in length. The latter are precursors of six different pre-miRN As. The *mir-17-92* gene cluster was used to create lentiviral vectors that direct the synthesis of four HIV1-specific miRN As. Sequences encoding pre-miRN As and containing 40 nucleotides on each side of the “loop-stem” structure were obtained from the gene cluster and were incorporated into the vector. Sequences of the guide strands of the future miRN As were replaced with segments specific with respect to HIV-1 (*Fig. 4C*) [69]. Due consideration was given to such features of the original structure of miRN As as mismatches and thermodynamic stability during the replacement of the sequences of the guide strand.

**The use of small interfering RNA**

Approaches for the clinical application of small interfering RN As are currently being developed. Dozens of siRN A-based medicinal agents designed to treat different kinds of diseases are currently undergoing clinical trials. Only one drug, which is based on the lentiviral delivery of shRN A, has been tested thus far. The use of lentiviral vectors directing the synthesis of shRN As is constrained by the fact that they are relatively unsafe. This is attributed to possible nonspecific responses, which can be caused by shRN A expression in cells and the probable insertional mutagenesis. However, the use of lentiviral vectors for siRN A delivery has a number of significant advantages. They can be used to achieve stable and prolonged shRN A synthesis in dividing and nondividing cells, making their application rather promising for the treatment of chronic diseases.

The mechanism of RN A interference is a component of the antiviral defense system of the organism; therefore, the use of RN A interference in chronic viral infections [59, 70, 71] (including the diseases caused by the hepatitis B and C and HIV-1 viruses) is of considerable interest. However, the use of RN A interference may result in the emergence of resistant forms of the virus, which limits the application of this method [72]. Contemporary methods enable the creation of lentiviral vectors that can simultaneously encode three or four shRN As which are specific with respect to various viral genes. This can significantly reduce the probability of emergence of resistant forms of the virus. Existing methods of siRN A delivery to T cells and macrophages (HIV-1 targets) are inefficient. The use of lentiviral vectors can be an efficient approach to introducing siRNAs into cells targeted by HIV-1. However, in the case of lentiviral vectors directing the synthesis of shRN As, which are specific with respect to viral genes, reduction in the efficiency of lentiviral particles and their titer is possible [73]. Thus, point mutations that do not affect the synthesis of the proteins required for the assembly of viral particles are introduced into the genes used in the packaging system. Selection of these mutations complicates the process of vector construction, especially if shRN As are selected for the conserved HIV-1 sites. After the infection with HIV-1, the viral envelope protein binds to the CD4^+^ receptor exposed on the surface of the target cells; the virus uses the CCR 5 cell receptor as a co-receptor. It has been demonstrated that homozygous deletion of the human *CCR5 *gene renders cells resistant to the HIV-1 infection, and the mutation apparently has almost no effect on the normal functioning of the cells [74]. It was demonstrated that shRN Amediated suppression of the CCR 5 receptor expression also renders cells resistant to infection by the virus *in vitro *and *in vivo *[75–78]. Several proteins whose functions are not essential to T cells or macrophages and which play an important role in the life cycle of HIV-1 have been identified [79].

The optimal approach is to obtain HIV-1-resistant T cells and macrophages from their common progenitors. To achieve this objective, transduction of early hematopoietic precursor cells was carried out using lentiviral vectors that direct the synthesis of shRN As that are specific with respect to the CCR 5 or CXCR -4 gene. The descendants of these cells (T cells and macrophages) acquired resistance to the virus [80 – 82]. There is an approach that enables the expression of shRN A, along with the other genes. The lentiviral vector that was successfully used to provide the synthesis of a false target for the viral TAT protein in addition to the expression of shRN A specific with respect to the general region of the *tat/rev *genes is an example of the latter concept. This false target impedes the action of the TAT protein and synthesis of ribozyme, which is specific with respect to the CCR 5 receptor [83, 84]. The efficiency of this vector was tested on humanized mice; stable inhibition of HIV-1 at different stages of the life cycle was achieved using this vector [85]. Clinical trials demonstrated the safety of using this vector in autologous bone marrow transplants in patients with HIV- 1 and lymphoma. The patients with lymphoma at the remission stage, which resulted from a conventional treatment regimen, exhibited no side effects associated with the introduction of shRN As. A detectable level of shRN A expression persisted in patients for 24 months.

**Fig. 5:**
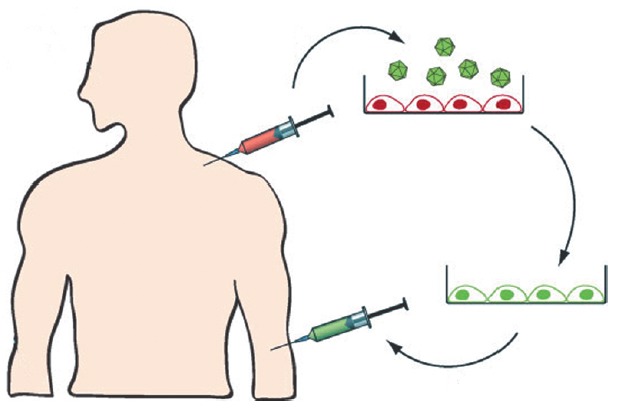
Ex vivo transduction of bone marrow cells. During autologous transplantation bone marrow cells are transduced with lentiviral vectors that direct the synthesis of shRNAs. Transduced cells are then administered to the patient following radiation therapy [84]

Malignant tumors develop as a result of mutations leading to an abnormal expression of the genes that stimulate cell proliferation and impairing apoptosis. RN A interference is a useful tool for modulating gene expression. It is considered that methods based on the principle of RN A interference can be of considerable interest in the treatment of tumors. The classical approaches to the therapy of malignant diseases are characterized by a number of significant deficiencies associated with the nonspecificity of their action. The use of RN A interference enables to exert a specific effect on oncogenes at a relatively low cost. A total of 10 siRN A-based drugs are currently undergoing clinical trials. The main obstacle associated with the use of RN A interference in treating malignant diseases is the imperfections of the methods for siRN A delivery to tumor cells. One of the most convenient and efficient gene transfer systems is based on the use of lentiviral vectors. These systems enable a highly specific integration of sequences encoding shRN A into the target cell’s genome. Methods for pseudotyping lentiviral particles are being developed and tissue-specific promoters are used in order to achieve this objective. Systems with multiplex shRN A expression show promise as well. Multiplex shRN A expression enables specific inhibition of multiple genes involved in tumor development. The use of multiple shRN As specific to different regions of the same activated oncogene makes it possible to improve the efficiency of these systems [86].

Many human miRN As are capable of inhibiting the growth of malignant tumors [87, 88]. Thereby, some of the research teams are working on the use of miRNAs for treating malignant tumors whose cells are characterized by a lower expression of oncosuppressor miRN As. It was demonstrated that restoration of the expression of oncosuppressor miRN A decreases cell growth in patients suffering from non-small-cell lung carcinoma, breast cancer, liver cancer, and chronic lymphocytic leukemia [89–92]. However, the inefficiency of *in vivo *transduction still remains the major problem when using lentiviral vectors as the main form of therapy. Leukemia therapy is considered to be the most suitable area for the use of lentiviral vectors that direct shRN A synthesis (*Fig. 5*). Autologous transplantation of hematopoietic cells transduced with a lentiviral vector which is specific with respect to one or several activated oncogenes can also be promising. The safety of this approach was demonstrated using lentiviral vectors that direct the synthesis of shRN As capable of inhibiting HIV-1 [93].

The search for new target genes that are involved in tumor development is also regarded as a promising application for shRN As. Nowadays, gene expression profiles in cancer cells are being actively studied. This has already enabled the discovery of several genes whose increased expression is associated with specific types of tumors. Vast libraries of lentiviral shRN A-based vectors enabling the search for the genes that are considered promising for the development of novel chemotherapeutic agents have been created [94, 95]. The shRN Ainduced inhibition of oncogene expression allows one to assess the contribution of these genes to the maintenance of the malignant status of tumor cells. A similar approach was used to transduce cells derived from a patient with acute myeloid leukemia using lentiviral vectors that direct shRN A synthesis, which are specific for *c-kit *and *AML1-ETO *oncogenes. The system was successfully used to investigate the inhibitory action of binase on the tyrosine kinase KIT receptor [96].

It is thought that shRN A can be successfully used in the gene therapy of neurodegenerative diseases, such as the Alzheimer’s, Parkinson’s and Huntington’s diseases. Their application is considered to be extremely promising, since lentiviruses are capable of overcoming the blood-brain barrier and infecting cells of the central nervous system (CN S). Lentiviral vectors enable to achieve a stable shRN A synthesis, which can be extremely important in the treatment of these chronic diseases. Pseudotyping of lentiviral vectors using the rabies virus envelope protein can increase efficiency in the infection of CN S cells.

## Abbreviations

siRNA: small interfering RNA
miRNA: microRNA
RISC: RNA-induced silencing complex
shRNA: small hairpin RNA
dsRNA: double-stranded RNA
HIV-1: human immunodeficiency virus type I
VSV-G: G protein of vesicular stomatitis virus
CMV: cytomegalovirus
H1, U6: DNA polymerase III promoters
